# Cutaneous stimulation at the ankle: a differential effect on proprioceptive postural control according to the participants’ preferred sensory strategy

**DOI:** 10.1186/s13047-016-0140-y

**Published:** 2016-03-08

**Authors:** Sébastien Pavailler, Frédérique Hintzy, Nicolas Horvais, Nicolas Forestier

**Affiliations:** Laboratoire Interuniversitaire de Biologie de la Motricité, Université de Savoie, UFR SceM - Technolac, 73376 Le Bourget du Lac, France; Salomon SAS, Amer Sports Footwear Innovation and Sport Science Lab, 14 chemin des Croiselets, 74996 Annecy, Cedex 9 France

**Keywords:** Vibration, Functional test, Relative proprioceptive weighting

## Abstract

**Background:**

Ankle movements can be partially encoded by cutaneous afferents. However, little is known about the central integration of these cutaneous signals, and whether individual differences exist in this integration. The aim of this study was to determine whether the effect of cutaneous stimulation at the ankle would differ depending on the participants’ preferred sensory strategy appraised by relative proprioceptive weighting (RPw).

**Methods:**

Forty-seven active young individuals free of lower-limb injury stood on a force platform either barefoot or wearing a custom-designed bootee. Vibrations (60 Hz, 0.5 mm) were applied either to the peroneal tendons or to the lumbar paraspinal muscles.

**Results:**

The barefoot RPw was strongly negatively correlated to the absolute change in RPw measured in the bootee condition (*r* = −0.81, *P* < 0.001). Participants were then grouped depending on their barefoot RPw value. The RPw was significantly higher in the bootee condition than in the barefoot condition only for participants with low barefoot RPw.

**Conclusions:**

The external cutaneous stimulation given by the bootee increased the weight of ankle proprioceptive signals only for participants with low barefoot RPw. This result confirmed that optimization of the ankle proprioceptive signals provided by cutaneous afferent stimulation has a differential effect depending on the participants’ preferred sensory strategy.

**Electronic supplementary material:**

The online version of this article (doi:10.1186/s13047-016-0140-y) contains supplementary material, which is available to authorized users.

## Background

Proprioception is usually defined as the ability to determine the position and velocity of one body segment with respect to the other [1, 2]. Proprioceptive signals are essential for movement regulation and postural control, as evidenced by studies on deafferented patients [3–6]. In consequence, a wide variety of external devices such as joint sleeves or braces, compressive stockings, and taping are currently designed to enhance proprioceptive signals in order to improve neuromuscular control. Proprioceptive signals arise from different mechanoreceptors located in the muscles, the articular and periarticular structures (capsule, ligaments, tendons), as well as in the skin. Because proprioception involves numerous physiological structures, from mechanoreceptors to cortical areas, its assessment is difficult. In the literature, clinical tests are the gold standard for proprioceptive evaluation. These test modalities are well documented [1, 2, 7] and basically consist of determining proprioceptive acuity by means of position replication tasks (matching tasks) or movement detection tasks. The principle of proprioceptive enhancement by an external device is based mainly on skin stretch and compression associated with an increase in cutaneous afferent stimulation, as movement can be partly encoded by this type of afferent [8, 9]. However, clinical testing of the aforementioned proprioceptive devices has given controversial results. Some studies showed that proprioceptive acuity at the knee [10, 11] or ankle [12, 13] was not enhanced by the application of an external cutaneous stimulation, whereas other studies found a positive effect of such devices on knee [14, 15] or ankle proprioception [16–21].

This heterogeneity may be due to confounding factors involved in proprioception. Three main factors can be identified in the literature. First, fatigue has been shown to alter proprioception at the ankle [22]. It was proposed that as fatigue increases the fusimotor activity because of afferents III and IV discharge, noise may be added to the system, thus leading to an altered proprioception. Numerous studies have also demonstrated that participants with chronic ankle instability showed impaired proprioception [23]. When an ankle sprain occurs, lateral ligamentous structures are disrupted. As these structures are highly innervated with mechanoreceptors, it is believed that these sensory receptors are also disrupted during the sprain, which lead to an altered proprioception [24]. Finally, because the number and size of proprioceptive receptors decrease with age, proprioceptive acuity has been shown to be lower in older people compared to younger people [25, 26]. Therefore, the positive effect of an external cutaneous stimulation observed in fatigued participants [15, 19], participants with chronic ankle instability [13, 17, 21] or older people [26] could be interpreted as a compensation of their poor baseline proprioceptive acuity. Yet, results are still heterogeneous even when focusing on studies involving young, healthy, unfatigued participants. Interestingly, two studies used the baseline proprioceptive acuity to specify the effects of external cutaneous stimulation on proprioception. In both experiments, healthy young participants were separated according to their barefoot proprioceptive acuity into a high and low acuity group [27, 28]. The results showed a positive effect of the external cutaneous stimulation on joint position sense only in the low acuity group, suggesting that individuals with low proprioception could more likely benefit from external cutaneous stimulation than people with normal proprioception. However, the methodological procedures used in the aforementioned studies are generally based on position replication tasks or movement detection tasks, which mainly reflect the participants’ analytical integration of proprioceptive signals. During the execution of natural or sport movements, the integration of proprioceptive signals refers to different and more complex nervous mechanisms, consisting of a sensory weighting process. Peterka [29] developed a so-called independent channel model that explains this weighting process. The model is based on three channels (visual, vestibular, and ankle proprioception) that are weighted independently as a function of the availability and accuracy of information. Some authors such as Isableu and Vuillerme [30] have used postural tasks to assess this sensory weighting process. These authors measured postural sway in healthy young participants on a firm support and on foam in order to alter proprioceptive signals originating from the ankle and foot sole. Their results demonstrated that the less the participants swayed on the firm support, the more they swayed on the foam, showing that the proprioceptive alteration given by the foam had a greater destabilizing effect in participants who were the most stable on the firm support. Similarly, Kluzik et al. [31] showed that some participants leaned forward after they stood on a toes-up inclined surface, whereas other participants remained relatively aligned when facing the same postural perturbation.

These two studies suggest that individuals seem to control standing balance quite differently. Participants seem to have individually preferred sensory strategies, meaning that each individual assigns different weights to each sensory signal available. Hence, some participants, also called *support-dependent*, appear to assign a high weight to ankle proprioceptive signals and control standing balance by regulating ankle angular variations with respect to the support. Conversely, others, also called *gravity-dependent*, may assign a high weight to graviceptive signals and control standing balance by exploring the gravity-inertial environment. Consequently, support-dependent participants are more perturbed by alterations of the support than the gravity-dependent participants. Brumagne et al. [32] proposed an alternative method based on muscular vibration to examine the sensory strategy during a postural task. The analysis of the disturbance induced by muscle vibration allows the determination of the relative proprioceptive weighting (RPw) index that represents the weight assigned to ankle proprioceptive signals to regulate standing balance. With this calculation, Brumagne et al. [32] showed that healthy participants exhibited lower RPw on foam than on the firm support. This indicates that the support characteristics may have an influence on the RPw depending on the participants’ preferred sensory strategy. As a consequence, it can be assumed that a device designed to optimize the ankle cutaneous proprioceptive signals would have a differential effect depending on the participants’ preferred sensory strategies. The aim of the present study was to determine whether the effect of a bootee designed to enhance ankle cutaneous proprioceptive signals by stretching and compressing the ankle skin would be different according to the participants’ preferred sensory strategy. To this aim, the RPw was calculated in healthy participants standing on a firm platform either barefoot or wearing the bootee. It was hypothesized that the bootee would increase the RPw only in participants who demonstrated a low barefoot RPw, that is, gravity-dependent participants.

## Methods

### Participants

Forty-seven healthy young individuals (41 males, six females) volunteered to participate in this study (age, 26.0 ± 7.4 years; height 1.77 ± 0.08 m; weight 70.3 ± 10.3 kg). They all reported regular physical activity (8.0 ± 5.5 h per week). Prior to the experiment, inclusion interviews were performed and individuals with a history of lower limb surgery, recurrent low back pain, neurological or balance disorders, a recent (<1 year) ankle sprain or regular use of ankle orthotics, a brace, or tape were excluded. The study was approved by the local research ethics committee, and informed consent was obtained from all participants included in the study in compliance with the Declaration of Helsinki (1964) for experimentation on humans.

### Protocol

**Fig. 1:**
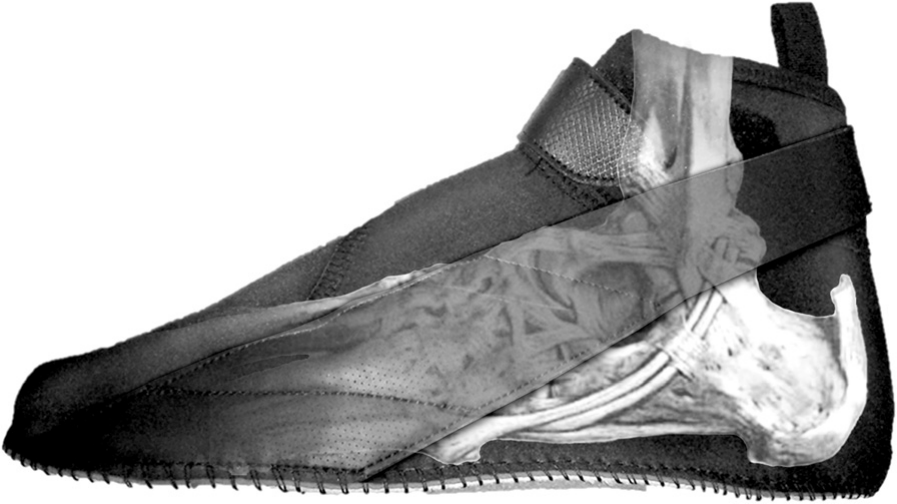
Picture of the bootee used in the experiment, superimposed on the foot anatomical structures. The bootee consists of a soft slipper including a tightening strap around the malleoli. The strap is connected to a lateral band crossing the ankle joint and following the main physiological structures (peroneal tendons, lateral hindfoot ligament). When movements occur at the hindfoot, a tension force is created in the band, which stretches and compresses the skin underneath

Two footwear conditions were used: an upright standing position on a stable platform (1) barefoot (BF condition) and (2) with a specifically designed bootee (BT condition). The BF condition was considered as baseline for all analysis. Figure 1 shows a picture of the bootee, which was constructed following Wilkerson’s recommendations [33] on external ankle devices. Both footwear conditions consisted of two trials during which vibration was applied either to the peroneal tendons or the lumbar paraspinal muscles, for a total of four trials per participant [34]. A limited number of trials was used to prevent the participants from any habituation effect [35]. The order of the trials for footwear and vibration conditions were randomized. All trials lasted 40 s, during which the participants were asked to stand on the force plate, the arms loosely hanging along the body. They were instructed to keep their eyes closed and to remain as immobile and relaxed as possible in the upright standing posture. The heels were 10 cm apart with the forefeet in a free splayed-out position. Once the position was chosen it was outlined on the platform for consistency across trials. The participants wore shorts and a T-shirt during the measurements.

### Muscle vibration

Mechanical vibration was used to stimulate proprioceptive receptors, in order to induce a perturbation in the standing posture and highlight the participants’ sensory strategy, potentially revealing an effect of the bootee that could not have been seen in an unperturbed system [36]. Two muscle vibrators (VB 115, Techno Concept, Mane, France) were held in place with rubber bands and bilaterally positioned over the peroneal tendons (2 cm above and behind the lateral malleoli) or lumbar paraspinal muscles. It was previously demonstrated that vibration applied to a muscle or a tendon may stimulate muscle spindles [37, 38] and tendon receptors [37]. Moreover, since vibration was elicited by vibrators placed on the skin, cutaneous mechanoreceptors may also have been stimulated [39]. Therefore, it can be assumed that all these types of receptors were stimulated in the present study. Peroneal and lumbar paraspinal muscles were selected to represent the muscles used in ankle and multisegmental postural strategy, respectively [40]. Muscle vibration was initiated 10 s after the start of the trial for 20 s. Activation and deactivation of the vibrators were manually controlled. The frequency of vibration was set at 60 Hz and the amplitude was approximately 0.5 mm. These vibration characteristics were chosen to induce maximal illusory joint movement and were demonstrated to induce a significant muscle lengthening illusion in healthy individuals [41]. Moreover, when peroneal tendons are vibrated in a healthy participant, postural sway in the backward direction is expected [34]. When lumbar paraspinal muscles are vibrated, a healthy participant is expected to show postural sway in the forward direction [32, 40].

### Postural measurements

Postural sway characteristics were measured using an eight-sensor force plate (Satel, Blagnac, France) and specifically dedicated software (Satel). Data were sampled at 50 Hz and included center of pressure (COP) coordinates in the anteroposterior and mediolateral direction. Only data in the anteroposterior direction was used for further analysis.

### Data reduction and statistical analysis

The mean values of the COP position in the anteroposterior direction during the muscle vibration trials were calculated over two periods: the 10 s preceding and the 20 s during muscle vibration. The last 10 s were not analyzed because they corresponded to the participant’s adaptation to the cessation of vibration. The proprioceptive postural control strategy or relative proprioceptive weighting (RPw) was appraised using eq. (1) [32]:

1 $$ RPw = \frac{\left|CO{P}_{vibrPER} - CO{P}_{base}\right|}{\left(\left|CO{P}_{vibrPER} - CO{P}_{base}\right| + \left|CO{P}_{vibrPS} - CO{P}_{base}\right|\right)}\times 100 $$

Where *COP* is the absolute value of the mean anteroposterior COP position, in the 10 s preceding vibration (*COP*_*base*_), during peroneal tendon vibration (*COP*_*vibrPER*_), and during paraspinal muscle vibration (*COP*_*vibrPS*_). This RPw (from 0 to 100) reflects the weight attributed to ankle proprioceptive signals in controlling standing balance, a high value indicating a high weight. Normality and homoscedasticity of all data sets were checked using a Shapiro–Wilk test and the Fisher F test, respectively. As recent studies suggested differences between men and women regarding balance control [42] and proprioception [43], a 2×2 ANOVA (two gender x two conditions) was performed on the RPw index to ensure there was no bias in lumping women and men together. A repeated-measure *t*-test was applied to the data of the BF and BT footwear conditions. The Pearson correlation coefficient (r) was calculated to assess whether the individual RPw differences between BT and BF footwear conditions were associated with the BF RPw. The significance level was set at *P* < 0.05.

Usually, participants are split into high and low proprioceptive acuity groups on the basis of their mean baseline (measured in the BF condition) proprioceptive acuity [27, 28]. In the present study the participants were divided into groups based on the standard error of measurement (SEM) of the RPw. Because the SEM represents the typical error that can be attributed to the measurement procedures, every individual change between two RPw values of less than 1 SEM can be attributed to this error. The 1 SEM value can be considered as the minimum difference between bootee RPw and barefoot RPw that can be attributed to wearing the bootee. Using the regression line of the difference between bootee RPw and barefoot RPw vs. the participant’s barefoot RPw (Fig. 1, panel b), it was possible to calculate a barefoot RPw threshold corresponding to the 1-SEM value (see the graphical representation of this threshold in Fig. 1, panel b).

To this aim, 21 of the 47 participants performed the two BF condition trials a second time in a separate testing session. The standard deviation of the individual’s differences between the two testing sessions was calculated and divided by the square root of 2 to obtain the SEM [44]. The RPw threshold was calculated as described above and used to assign participants to a low barefoot RPw group (LOW) or to a high barefoot RPw group (HIGH). RPw values were compared using a 2 × 2 (two groups × two footwear conditions) repeated-measures ANOVA, with a significance level set at *P* < 0.05. Post-hoc comparisons (Sheffe’s test) were performed whenever necessary.

## Results

There was no interaction between gender and condition (*F(1,90)* = 0.214, *P* = 0.645) nor gender main effect (*F(1,90)* = 0.494, *P* = 0.484) on the RPw index, and consequently results for women and men were lumped together.

**Fig. 2:**
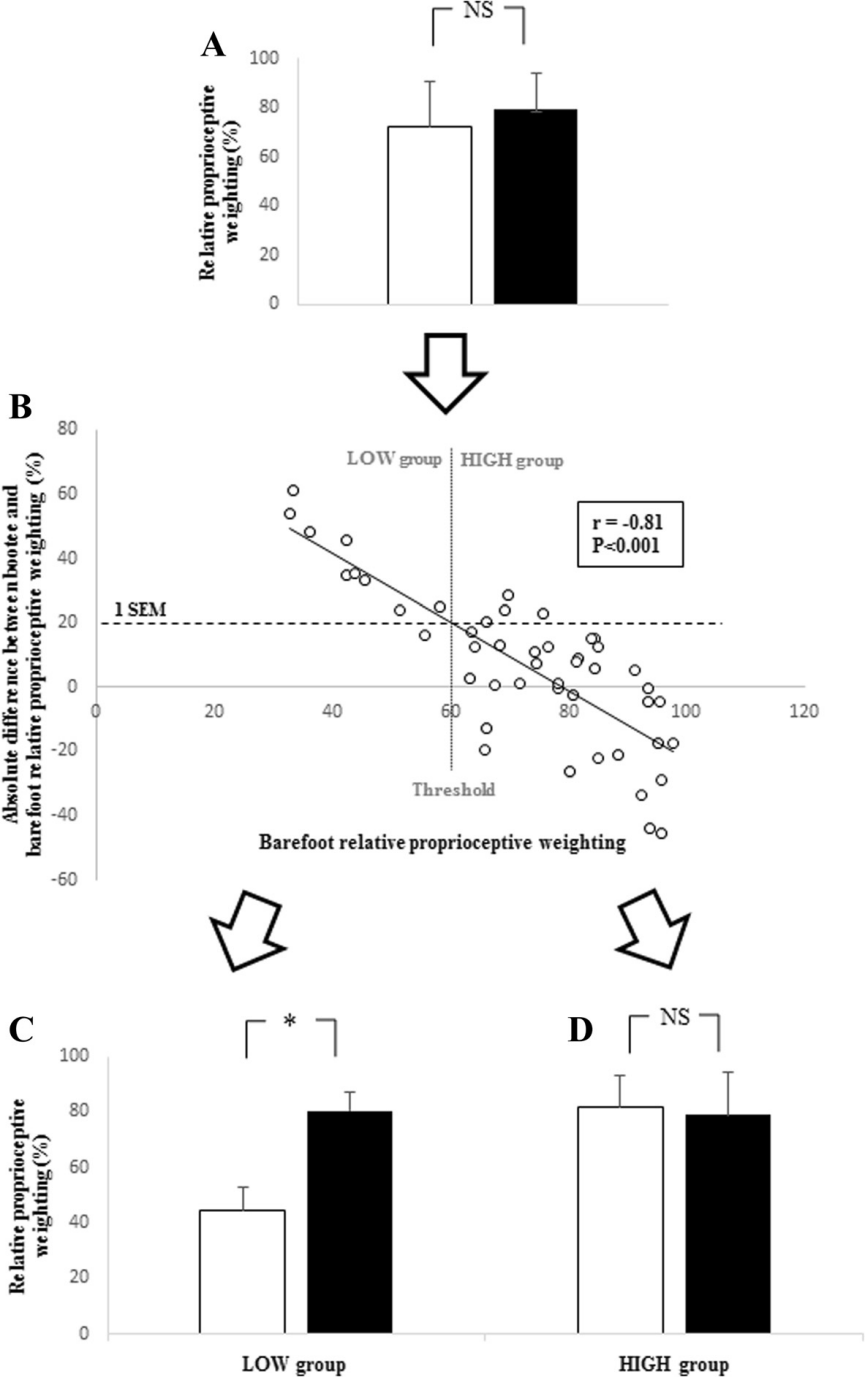
Main results of the experiment. Panel **a** Means and standard deviation of the relative proprioceptive weighting pooled for all the participants in the barefoot condition (white bar) and the bootee condition (black bar). Panel **b** Scatter plots of the baseline relative proprioceptive weighting in the barefoot condition vs. the absolute change in relative proprioceptive weighting in the bootee condition, and subsequent division between high and low baseline relative proprioceptive weighting group (HIGH and LOW). The threshold corresponds to the X axis value of the intersection between the 1 SEM line (dotted line) and the regression line (black line). Panel **c** and (**d**) Means and standard deviation of the relative proprioceptive weighting for the low baseline relative proprioceptive weighting group (**c**) and the high baseline relative proprioceptive weighting group (**d**) in the barefoot condition (white bars) and the bootee condition (black bars). *: statistical difference between the datasets (*P* < 0.05), NS: not significant

Analysis of the RPw values showed no significant difference between the BT and the BF footwear conditions (BT: 79.5 ± 14.2 % CI 95 % [75.5, 83.6] vs. BF: 72.6 ± 18.3 % CI 95 % [67.4, 77.9], *P* = 0.06, Fig. 2, panel a). The associated Cohen’s *d* effect size was medium (*d* = 0.43, CI 95 % [-3.70, 2.85]). The barefoot RPw was strongly negatively correlated to the difference between RPw in the BT and BF footwear conditions (*r* = – 0.81, *P* < 0.001, Fig. 2, panel b).

The SEM in the BF condition was 19.7 %, and corresponded to a barefoot RPw threshold of 60.6 % (Fig. 2, panel b). Thus the LOW group included the ten participants with a barefoot RPw less than 60.6 %, and the HIGH group included the 37 participants with a barefoot RPw greater than 60.6 %.

The ANOVA revealed a significant interaction between group and footwear condition on RPw (*F(1, 90)* = 38.210, *P* < 0.001). The post-hoc analysis showed that RPw was significantly higher in the BT footwear condition (82.0 ± 6.78 %, CI 95 % [77.8, 86.2]) than in the BF footwear condition (44.2 ± 8.86 %, CI 95 % [38.7, 49.7]) for the LOW group (Fig. 2, panel c). The associated effect size was very large (*d* = 5.05, CI 95 % [1.77, 8.33]). For the HIGH group (Fig. 2, panel d), no significant differences were observed between the footwear conditions (BT: 78.9 ± 15.6 % CI 95 % [73.8, 83.9] vs. BF: 80.3 ± 11.0 % CI 95 % [76.8, 83.9]). The associated effect size was negligible (*d* = 0.11, CI 95 % [-2.93, 3.14]). Data sets supporting these results are available as Additional file 1.

## Discussion

The aim of this study was to determine whether the effect of a bootee designed to provide cutaneous stimulation at the ankle would differ according to the participants’ preferred sensory strategy. When all participants were considered, the results showed that the cutaneous stimulation induced by the bootee was not associated with an increase in the weight of ankle proprioceptive signals. However, when the overall population was separated into individuals allocating a high weight to ankle proprioceptive signals and individuals allocating a high weight to graviceptive signals on the basis of the relative proprioceptive weighting (RPw), the results indicated that the application of the bootee had a differential effect depending on the participants’ preferred sensory strategy. More precisely, the external cutaneous stimulation increased the weight of ankle proprioceptive signals only in participants with low barefoot RPw, that is, in gravity-dependent participants.

To our knowledge, this study is the first to propose a clear identification of the participants’ preferred sensory strategy, on the basis of a functional index (RPw). Isableu and Vuillerme [30] and Kluzik et al. [31] had already suggested the existence of these strategies, but using indirect evidence. In the present study we used a muscle vibration technique that had been proven to clearly interrogate the proprioceptive system [45, 46] and allow determination of the weighting of ankle proprioceptive signals [32, 34]. In that way we addressed more directly the preferred sensory strategies and proposed a clear distinction between them.

Moreover this study clarified the effect of an ankle external cutaneous stimulation on postural control. First, we made sure to control some confounding factors known to have an effect on baseline proprioception (fatigue, age, chronic ankle instability). Then, as we precisely determined the participants’ preferred sensory strategies, we were able to identify the population that was effected by an external stimulation. The specific increase in RPw observed for the gravity-dependent participants is in line with the results obtained by Cameron et al. [27] and You et al. [28]. These authors used matching tasks at the leg [27] or at the ankle [28] to assess the effect of cutaneous afferent stimulation (by close fitting shorts or an ankle pressure cuff) on proprioception. They showed that cutaneous afferent stimulation increased the matching task performance only in participants with a poor baseline proprioceptive level. However, while these two studies reflected the participants’ analytical integration of the proprioceptive signals during a simple single-joint movement, the present study using a postural, multisegment and much more challenging task reflected a more complex nervous mechanism, which consists in a sensory weighting of the various signals available for posture regulation. These results have important implications for the testing of external ankle devices. As shown in the present study, the effect of the cutaneous stimulation may be different from one participant to another. Thus, if one aims at demonstrating any ankle proprioceptive optimization, we would recommend to only include gravity-dependent participants. Inclusion procedures should be based on the vibration techniques used in the present study as they allow determination of each participant’s preferred sensory strategy by mean of the RPw index. This index could be further used to specifically adapt training or rehabilitation programs as a function of the participant’s profile.

It could be argued that the individual differences observed may be attributed to anatomical discrepancies located around the foot and the ankle rather than to sensory strategies. Since the foot and the ankle morphology was not controlled, wearing the bootee could have induced individual differences in cutaneous tissues deformation and, as a result, in cutaneous proprioceptive signals. However, given the soft material of which the bootee was made and the presence of reinforcements only around the malleoli, it can be reasonably assumed that the deformation was limited and reproducible. One other limitation to the present study could be the lack of control of instantaneous COP position when delivering vibrations. Vibrations were delivered at the same time point for every participant, but we did not have any real time feedback of the COP anteroposterior position at this particular time point. Consequently, discrepancies may have occurred between participants, potentially leading to individual differences in the postural responses to vibration. Indeed, a recent study showed that postural responses to ankle tendons vibration were dependent on the direction of postural leaning [47]. These responses were augmented in backward leaning and attenuated in forward leaning. However, the leaning amplitudes used in Kanakis’ study [47] were quite large as the participants were asked to transfer 80 % of their body weight backward or forward. It may be assumed that in a conventional postural task like the one used in the present study, the leaning amplitudes (or COP excursions) are lower and the alterations of postural responses to vibration are limited.

The main explanation of the present findings is based on the independent channel model developed by Peterka [29]. According to this model sensory signals are weighted independently as a function of the availability and accuracy of the information. It can be assumed that if one of the channels is not available, its weight is set to zero and the system is only based on the two remaining channels. This was certainly the case for all participants in the present study because their eyes were closed during all the tests and, as a result, visual channel weight may have been set to zero. In this context, accuracy is determined by comparing sensory feedback of the proprioceptive channel with an internal reference based on a copy of motor command (efference copy). The lower the deviation from the reference (what can be called the error signal), the higher the weight assigned to the channel. It can be speculated that the individuals’ preferred sensory strategies arise from discrepancies in this weighting process. A gain could be applied to the error signal when assessing the accuracy of the channel, thus leading to a preferred weighting of a particular channel. This could be the reason why, in an eyes-closed postural task, some individuals might apply a low error gain to the ankle proprioceptive channel, which leads to a heavy weight on this channel and a high RPw, that is, for support-dependent participants. In contrast, other individuals might apply a high error gain to the ankle proprioceptive channel, which leads to a low weight on this channel and a low RPw (e.g., gravity-dependent participants). It is notable that only the gravity-dependent participants changed their sensory strategy when wearing the bootee.

It is likely that the support-dependent participants had sufficient sensory feedback from the plantar sole and the ankle joint such that the extra information contributed by the bootee was not used. On the other hand, the gravity-dependent participants may use the extra proprioceptive information from the bootee and thus reweight the proprioceptive signals coming from their ankles. This indicates that extra information originating from ankle skin might lower the potentially high error gain applied to the ankle proprioceptive channel and, consequently, increase the weight assigned to this channel. This reweighting may reflect the simultaneous and combined integration of cutaneous and muscle afferent signals. It is established that cutaneous and muscle afferents at the ankle provide much the same information on movement direction and velocity [8, 9]. Therefore, both of these signals could be used by the central nervous system in order to control movement.

However, because these studies used microneurographical techniques at a peripheral level, no indications on how these signals are centrally processed and weighted for perceptual purposes and movement control were given. Several studies investigated the integration of cutaneous and muscular proprioceptive signals at the fingers, elbow, and knee [48, 49] by applying stretching to the skin and vibration to the muscles. The results showed that muscle vibration alone produced large and global illusory movements, whereas skin stretch alone produced little illusory movement. However, skin stretch focused and increased movement illusion when applied simultaneously with muscle vibration. This suggests that execution of movement could be assisted by a cutaneous stimulation close to the movement location. It is useful to note that for some participants, skin stretch did not increase movement illusion or even decreased it. In the present study, the application of the bootee may have caused a cutaneous stimulation similar to the skin stretch used in Collins et al.’s studies [48, 49], leading to a reweighting of the ankle proprioceptive channel. It can be assumed that when muscle vibration was applied, the cutaneous stimulation provided by the bootee may have increased movement illusion in gravity-dependent participants, while no effects were induced in support-dependent participants. Finally, the results obtained support the main idea that devices designed to provide a joint external cutaneous stimulation may have a positive effect on the weight of proprioceptive signals by stimulating cutaneous afferents only for some individuals.

## Conclusion

To summarize, this study showed that the external cutaneous stimulation given by the bootee had an effect on the weight of ankle proprioceptive signals only in participants with a particular preferred sensory strategy, that is, gravity-dependent participants. It is proposed that this reweighting reflects the simultaneous and combined central integration of cutaneous and muscle afferent signals. Consequently, future testing of any ankle external device based on cutaneous stimulation (ankle sleeves or braces, compressive stockings, taping) should be conducted preferentially among those participants. This raises the necessity to perform inclusion procedures prior to testing. Further work is planned to examine the effect of an ankle external cutaneous stimulation in a more global situation than a postural task. The objective is to link the sensory reweighting brought by the cutaneous stimulation to more global biomechanical parameters. Based on the present results, only gravity-dependent participants will be included in this work.

### Ethics approval and consent to participate

The study was approved by the University of Savoy local research ethics committee, and informed consent was obtained from all participants included in the study in compliance with the Declaration of Helsinki (1964) for experimentation on humans.

### Consent for publication

Not applicable.

### Availability of data and materials

The datasets supporting the conclusions of this article are included within the article and its additional file.

## Additional file

Additional file 1: Data sets of the study. (XLSX 53 kb)

## Abbreviations

ANOVA: Analysis of Variance
BF: Barefoot condition
BT: Bootee condition
CI: Confidence interval
COP: Centre of pressure
HIGH: High barefoot RPw group
LOW: Low barefoot RPw group
RPw: Relative proprioceptive weighting
SEM: Standard Error of Measurement
